# A Low-Complexity Geometric Bilateration Method for Localization in Wireless Sensor Networks and Its Comparison with Least-Squares Methods

**DOI:** 10.3390/s120100839

**Published:** 2012-01-12

**Authors:** Juan Cota-Ruiz, Jose-Gerardo Rosiles, Ernesto Sifuentes, Pablo Rivas-Perea

**Affiliations:** 1 Department of Electrical and Computer Engineering, Autonomous University of Ciudad Juárez (UACJ), Ave. del Charro # 450 Nte. C.P.32310, Ciudad Juárez, Chihuahua, México; E-Mail: esifuent@uacj.mx; 2 Rosiles Consulting, El Paso, TX 79938, USA; E-Mail: rosiles@ieee.org; 3 Department of Computer Science, Baylor University, One Bear Place #97356, Waco, TX 76798, USA; E-Mail: Pablo Rivas Perea@baylor.edu

**Keywords:** distributed-localization, wireless sensor networks, Least Squares (LS), optimization, bilateration

## Abstract

This research presents a distributed and formula-based bilateration algorithm that can be used to provide initial set of locations. In this scheme each node uses distance estimates to anchors to solve a set of circle-circle intersection (CCI) problems, solved through a purely geometric formulation. The resulting CCIs are processed to pick those that cluster together and then take the average to produce an initial node location. The algorithm is compared in terms of accuracy and computational complexity with a Least-Squares localization algorithm, based on the Levenberg–Marquardt methodology. Results in accuracy *vs.* computational performance show that the bilateration algorithm is competitive compared with well known optimized localization algorithms.

## 1. Introduction

Recent advances in microelectronics have led to the development of autonomous tiny devices called sensor nodes. Such devices, in spite of their physical limitations, contain the essential components of a computer, such as memory, I/O ports, sensors, and wireless transceivers which are typically battery-powered. Once deployed (randomly or not) over a certain area, sensor nodes have the ability to be in touch via wireless communications with neighboring nodes forming a wireless sensor network (WSN). The great advantage of using WSNs is that they can be applied in important areas such as disaster and relief, military affairs, medical care, environmental monitoring, target tracking, and so on [1–3]. However, most of WSN applications are based on local events. This means that each sensor node needs to detect and share local phenomenons with neighboring nodes, implying that the location of such events (*i.e.*, sensor locations) are crucial for the WSN application. In this way, sensor self-positioning represents the first startup process in most WSN projects. It is well known that using a GPS in each sensor node represents the primary solution to infer position estimates. However, this option is not suitable to be considered in all nodes if parameters like size, price, and energy-consumption in a sensor node are of concern [4]. In order to optimize such parameters, a good option consists of reducing to a small fraction of sensors with GPS, and the remainder sensors (*i.e.*, unknown sensors), commonly above 90% of total deployed sensors, should use alternatives to estimate its own positions like radio-frequency transmissions or connectivity with neighboring sensors [5–7].

In order to provide position estimates many localization algorithms have been proposed, coming from different perspectives as described in [8,9]. Basically, localization algorithms can be categorized according to range-based *vs.* range-free methods, anchor-based *vs.* anchor-free models, and distributed *vs.* centralized processing [10,11]. Range-based methods consist of estimating node locations (using a localization algorithm) based on ranging information among sensor nodes. Range estimation between pairs of nodes is achieved using techniques of Time-of-Arrival (ToA), Receive-Signal-Strength (RSS), or Angle-of-Arrival (AoA) [12]. This approach has the disadvantage of requiring extra-hardware in each sensor board, increasing the cost per sensor. However, as far as is known, this approach provides the best cost-accuracy performance in localization algorithms. A less expensive but more inaccurate alternative consists of using just connectivity among sensor nodes to estimate node locations, called range-free [13]. On the other hand, if position estimates are obtained by considering absolute references (e.g., sensors with GPS or Anchors), the resulted position estimates (also with absolute positions) will be closely related to such reference positions, called an anchor-based model. By the contrary, if no reference positions are used to estimate locations, relative coordinates will be obtained, called an anchor-free model.

One of the most interesting and relevant aspects in WSN localization is associated with the way to compute the location of sensor nodes. For example, if all pairwise distance measurements among sensor nodes are sent to a central node to compute position estimates, the localization algorithm becomes centralized. This kind of central processing has the advantage of global mapping, but it has basically two important disadvantages which demerit its use in many cases when robustness and saving-energy have high priority in a WSN [14]. Some important centralized schemes are the next. In [15] an iterative descent procedure (*i.e.*, Gauss–Newton method) is used in a centralized way to solve the Non-Linear Least-Square (NLLS) problem. Another interesting centralized scheme was proposed in [16] where the WSN localization problem is modeled as linear or semidefinite program (SDP), and a convex optimization is used to solve problem.

In contrast, when each sensor node estimates its own location using available information of neighboring nodes (e.g., range, connectivity, location, *etc*.), the localization process becomes distributed. Distributed processing is much less energy consuming in WSNs than centralized processing because centralized schemes need to collect relevant information from all nodes in the network which implies re-transmissions in multi-hop environments. Also, distributed algorithms are tolerant to node failures due to node redundancy. Thus, basically a distributed algorithm allows robustness, saving-energy, and scalability [14,17,18], which overcomes the limitations imposed by the centralized approach. In [19], a robust least squares scheme (RLS) for multi-hop node localization is proposed. This approach reduces the effects of error propagation by introducing a regularization parameter in the covariance matrix. However, the computational cost to mitigate the adverse effects of error propagation is too high at energy-constrained nodes. Similarly, [20] proposes two weighted least squares techniques to gain robustness when a non-optimal propagation model is considered however they failed to introduce a covariance matrix in the localization process that can effectively decrease the computational complexity. On the other hand, the authors of [21] propose a Quality of Trilateration method (QoT) for node localization. This approach provides a quantitative evaluation of different geometric forms of trilaterations. However, it seems to be that the main idea of this methodology depends on the quality or resolution of geometric forms (*i.e.*, like image processing) which is impractical to be implemented in resource-constrained devices with limited memory and processing capabilities (*i.e.*, nodes).

In this paper, we analyze a range-based bilateration algorithm (BL) that can be used in a distributed way to provide initial estimates for unknown sensors in a wireless sensor network (our analysis consider that each unknown sensor can determine its initial position communicating directly with several anchors). In this case, each node uses a set of two anchors and their respective ranges at a time to solve a set of circle intersection problems using a geometric formulation. The solutions from these geometric problems are processed to pick those that cluster around the location estimate and then take the average to produce an initial node location. Finally, we present a computational/accuracy comparison between the BL algorithm, based on closed-formulas, and a classical Least Squares (LS) approach for localization, based on the iterative Levenberg–Marquardt algorithm (LM).

The outline of this paper is as follows. In Section 2 we examine a popular ranging technique for WSNs used in our simulations. In Section 3 we explore the localization problem from the Least Squares point of view. In Section 4 we analyze in detail the BL algorithm. In Sections 5 and 6 we evaluate the accuracy and computational-complexity performance respectively between the bilateration algorithm *vs.* LS schemes. Finally, we present our conclusions.

## 2. Ranging Techniques

This section presents a brief overview of an existing ranging technique used to estimate the true distance between two sensor nodes using power measurements, called Received Signal Strength (RSS). This technique is popular because sensor nodes do not require special hardware support to estimate distances. As a first approximation, considering the free space path loss model, the distance *d_ij_* between two sensors *s_i_* and *s_j_* can be estimated by assuming that the power signal decreases in a way that is inversely proportional to the square of the distance $\left(1/d_{\mathrm{ij}}^{2}\right)$. However, in real environments the signal power is attenuated by a factor *d*^−η*_p_*^. The path-loss factor η*_p_* is closely related to geometrical and environmental factors, and it varies from 2 to 4 for practical situations [22]. In noiseless environments the power signal traveling from a sensor *s_j_* to a sensor *s_i_* can be measured according to the relation [23]

(1) $$P_{\mathrm{ij}}=P_{0}\;{\left(\frac{d_{0}}{d_{\mathrm{ij}}}\right)}^{\eta_{p}}$$

where the path-loss factor (η*_p_*) depends directly on the environmental conditions. *P*_0_ is the received power at the short reference distance of *d*_0_ = 1*m* from the transmitter. Also, *P*_0_ can be computed by the Friis free space equation [24]. The log-distance path loss model

(2) $${\bar{P}}_{L}^{\left(ij\right)}(\mathrm{dB})=P_{0}-10\eta_{p}\text{log}\frac{d_{\mathrm{ij}}}{d_{0}}$$

measures the average large-scale path loss between sensors *s_i_* and *s_j_*. The actual path-loss (in dB) is a normally distributed random variable:

(3) $$P_{L}^{\left(\mathrm{ij}\right)}∼𝒩\left({\bar{P}}_{L}^{\left(\mathrm{ij}\right)},\sigma_{\mathrm{SH}}^{2}\right)$$

where σ*_SH_* is given in dB and reflects the degradations on signal propagation due to reflection, refraction, diffraction, and shadowing. It can be seen that the linear measurements and distance estimates have a log-normal distribution with a multiplicative effect on the measurements. The noisy range measurement *R_ij_* can be obtained from Equations (2) and (3) as

(4) $$R_{\mathrm{ij}}=10^{\frac{P_{0}-P_{L}^{\left(\mathrm{ij}\right)}}{10-\eta_{p}}}$$

## 3. Least-Squares Multilateration Localization Algorithms

In this section, we describe multilateration schemes that provide solutions to the Least-Square (LS) problem for location estimates using noisy ranging information derived from ToA or RSS ranging techniques. Consider a set of *N* wireless sensor nodes **S** = {*s*_1_, *s*_2_,..., *s_N_*}, randomly distributed over a 2-D region whose locations are unknown. We represent these unknown locations with vectors **z***_i_* = [*x_i_, y_i_*]*^T^*. Further, we assume the presence of a set **A** = {*a*_1_*,a*_2_*,...,a_M_*} of *M* reference or anchor nodes with known position **q***_j_* = [*x_j_, y_j_*]*T*. Anchor nodes, *a_i_*, are equipped with GPS or a similar scheme to self localize. Also, for practical situations *M ≪ N* with *M >* 2. We develop our discussion assuming a 2-D scenario, but it can be easily generalized to the 3-D case.

Moreover, we assume that any sensor can estimate pairwise ranges with its neighbors using time-of-arrival (ToA) or radio signal strength (RSS) techniques [24]. Denote the range estimate between the node *s_i_* and anchor *a_j_* as

(5) $$R_{\mathrm{ij}}=d_{\mathrm{ij}}+e_{\mathrm{ij}}$$

where *d_ij_* is the true distance between *a_j_* and *s_i_*, and *e_ij_* represents the measurement error introduced by environmental noise, propagation distortion, and the ranging technique. Then the solution to the localization problem for a node *s_i_* consists of minimizing the sum of certain weighted error-distance function *e_w_*(*·*) as follows:

(6) $$p_{i}=\underset{x}{\text{arg min}}\sum_{j=1}^{M}e_{w}\left(\left\|q_{j}-x\right\|-R_{\mathrm{ij}}\right)=\underset{x}{\text{arg min}}\;𝒡\;(x)$$

where **p***_i_* = [*x_i_,y_i_*]*^T^* represents the most likely position for the sensor *s_i_* that minimizes *𝒡*, ‖ · ‖ represents the Euclidean norm, and *e_w_*(*x*) represents a function that provides a specific weight to the argument *x* (*i.e.*, error distance). For example, the function *e*_2_(*x*) = (*x*)^2^, the LS formulation, is commonly used to solve Equation (6) due its tractability and efficiency in both mathematical and computational analysis. The LS problem can be solved either by closed-form solutions or by iterative methods. Next we describe both methodologies in detail.

### 3.1. Closed-Form LS Multilateration

Closed-Form methods have the advantage of fast time processing, which is useful for constrained devices (*i.e.*, motes) where the energy conservation represents one of the major concerns. However, this approach is also subject to inaccurate estimates due to noisy ranging measurements, so in most cases this approach is not a suitable option in real WSN scenarios where current ranging techniques are not able to provide the required accuracy on the ranging measurements. For example, Spherical Intersection (SX), Spherical Interpolation (SI), and Global Spherical Least Squares (GSLS) [25] can solve a nonlinear set of equations using closed-formulas. These approaches provide good accuracy in the estimated positions under conditions like small biases and small standard deviations, but they also provide meaningless estimates under noisy environments [26]. A more robust closed-form scheme consists of using the classical LS multilateration discussed next [19,27,28].

Consider that a sensor *s_i_* with Cartesian position **p***_i_* = [*x_i_, y_i_*]*^T^* has already estimated its range *R_ij_* to *M* anchors. For each anchor *a_j_* with position **q***_j_* = [*x_j_, y_j_*]*^T^*, an equation ${\left\|q_{j}-p_{i}\right\|}^{2}=R_{\mathrm{ij}}^{2}$ is generated as shown the next formulas:

(7) $$\begin{array}{ccc} {\left\|q_{1}-p_{i}\right\|}^{2}=R_{i1}^{2} & & {\left(x_{1}-x_{i}\right)}^{2}+{\left(y_{1}-y_{i}\right)}^{2}=R_{i1}^{2}\\ {\left\|q_{2}-p_{i}\right\|}^{2}=R_{i2}^{2} & \Leftrightarrow & {\left(x_{2}-x_{i}\right)}^{2}+{\left(y_{2}-y_{i}\right)}^{2}=R_{i2}^{2}\\ \vdots & & \vdots \\ {\left\|q_{M}-p_{i}\right\|}^{2}=R_{\mathrm{iM}}^{2} & & {\left(x_{M}-x_{i}\right)}^{2}+{\left(y_{M}-y_{i}\right)}^{2}=R_{iM}^{2} \end{array}$$

The system of Equations (7) can be linearized by subtracting the first equation (*j* = 1) from the last *M −* 1 equations arriving to a linear system that can be represented in a matrix form as

(8) $${\mathrm{Ap}}_{i}=b$$

where

(9) $$A=-2\;{\left[\begin{array}{cc} x_{2}-x_{1} & y_{2}-y_{1}\\ x_{3}-x_{1} & y_{3}-y_{1}\\ \vdots & \vdots \\ x_{M}-x_{1} & y_{M}-y_{1} \end{array}\right]}_{(M-1)\times 2}$$

(10) $$b={\left[\begin{array}{c} R_{i2}^{2}-R_{i1}^{2}+x_{1}^{2}+y_{1}^{2}-x_{2}^{2}-y_{2}^{2}\\ R_{i3}^{2}-R_{i1}^{2}+x_{1}^{2}+y_{1}^{2}-x_{3}^{2}-y_{3}^{2}\\ \vdots \\ R_{\mathrm{iM}}^{2}-R_{i1}^{2}+x_{1}^{2}+y_{1}^{2}-x_{M}^{2}-y_{M}^{2} \end{array}\right]}_{(M-1)\times 1}$$

Now the least squares solution to Equation (8) is to determine an estimate for **p***_i_* that minimizes

(11) $$\begin{array}{l} f\left(p_{i}\right)=\underset{p_{i}}{\text{min}}\left\{\frac{1}{2}{\left\|{\mathrm{Ap}}_{i}-b\right\|}^{2}\right\}\\ \;\;\;\;\;\;\;\;\;\;\;\;\;=\underset{p_{i}}{\text{min}}\left\{\frac{1}{2}{\left({\mathrm{Ap}}_{i}-b\right)}^{T}\left({\mathrm{Ap}}_{i}-b\right)\right\} \end{array}$$

After some manipulations we obtain the following:

(12) $$f\left(p_{i}\right)=\underset{p_{i}}{\text{min}}\left\{\frac{1}{2}p_{i}^{T}A^{T}{\mathrm{Ap}}_{i}-p_{i}^{T}A^{T}b+\frac{1}{2}b^{T}b\right\}$$

and the gradient of *f* at **p***_i_* is

(13) $$\nabla f\left(p_{i}\right)=A^{T}{\mathrm{Ap}}_{i}-A^{T}b=0$$

which provides the estimate (*i.e.*, normal equations) to Equation (8):

(14) $${\hat{p}}_{i}={\left(A^{T}A\right)}^{-1}A^{T}b$$

Solving for Equation (14) may not work properly if **A***^T^* **A** is close singular, so a recommended approach is to use a Tikhonov regularization as follows:

For *μ >* 0 (e.g., close to zero)

(15) $$\begin{array}{l} f_{\mu }\left(p_{i}\right)=\underset{p_{i}}{\text{min}}\left\{\frac{1}{2}{\left\|{\mathrm{Ap}}_{i}-b\right\|}^{2}+\frac{\mu }{2}\left\|p_{i}\right\|\right\}\\ \;\;\;\;\;\;\;\;\;\;\;\;\;=\underset{p_{i}}{\text{min}}\left\{\frac{1}{2}p_{i}^{T}A^{T}{\mathrm{Ap}}_{i}-p_{i}^{T}A^{T}b+\frac{1}{2}b^{T}b+\frac{\mu }{2}p_{i}^{T}p_{i}\right\} \end{array}$$

Then the gradient of *f_μ_* at **p***_i_* is

(16) $$\nabla f_{\mu }\left(p_{i}\right)=A^{T}{\mathrm{Ap}}_{i}-A^{T}b+\mu p_{i}=0$$

Factorizing we arrive to a robust estimate for the LS problem where the idea is to modify eigenvalues to avoid working with zero eigenvalues [19,29].

(17) $${\hat{p}}_{i}={\left(A^{T}A+\mu I\right)}^{-1}A^{T}b$$

### 3.2. Iterative LS Algorithms

Iterative methods are usually employed either when large-data set of information need to be processed or when an exact solution to a certain problem is not feasible (e.g., non-linear systems of equations) [30]. Optimization techniques represent a good alternative to solve such non-linear equations using an iterative procedure. Optimization algorithms that solve Non-Linear Least-Square (NLLS) problems (*i.e.*, the WSN localization problem) have been extensively proposed where the Newton or Quasi-Newton methods are iteratively used to minimizing some residuals [15,31,32]. The next paragraphs describe two well known iterative algorithms that are used to solve the NLLS problem: the Levenberg–Marquardt (LM) and the Trust-Region-Reflective (TRR).

Assuming that a node denoted **s***_i_*, with Cartesian position **p***_i_* = [*x_i_, y_i_*]*^T^*, estimates its distance *R_ij_* to *M* anchors denoted *a_j_*, with positions **q***_j_* = [*x_j_, y_j_*]*^T^*, with *j* = 1, ..., *M*. Consider the following residual error vector:

(18) $$R\left(p_{i}\right)=\left[\begin{array}{c} R_{i1}-\left\|p_{i}-q_{1}\right\|\\ R_{i2}-\left\|p_{i}-q_{2}\right\|\\ \vdots \\ R_{\mathrm{iM}}-\left\|p_{i}-q_{M}\right\| \end{array}\right]$$

Therefore, to find the more likely position of **p***_i_*, the program

(19) $$\underset{p_{i}}{\text{min}}\;f\;(p_{i})=\underset{p_{i}}{\text{min}}\;(\frac{1}{2}R{\left(p_{i}\right)}^{T}R\left(p_{i}\right))$$

is solved, which is the least squares problem.

To solve Equation (19) we employ the TRR algorithm and the LM algorithm. The TRR algorithm uses a sub-space trust-region method to minimize a function *f*(*x*). Here, approximations to *f* inside of a trust-region are iteratively required. The three main concerns in this algorithm are how to choose and compute the approximation to the function, how to choose and modify the trust region, and, finally, how to minimize over the sub-space trust-region. Even though the TRR algorithm provides an accurate solution for the WSN initial estimates, it is expensive (computationally speaking) for constrained sensor nodes [33].

On the other hand, the LM algorithm uses the search direction approach (a mix between the Gauss–Newton direction and the steepest descent direction) to find the solution to Equation (19). This algorithm outperforms the simple gradient descent methodology [34], and also it avoids dangerous operations with singular matrices as the pure Newton method does, so this methodology represents a good algorithm for comparison with the bilateration approach due to its robustness, speed, and accuracy [35]. Following the procedure presented in [29], Equation (19) can be solved by the Line Search Levenberg–Marquardt methodology shown in Algorithm 1, where ‖·‖ is the *ℓ*-2 norm, **I** is the identity matrix, *R_ij_* is the estimated distance between the mote *s_i_* and the anchor *a_j_*, **J**(**p***_k_*) represents the Jacobian of **R**(**p***_k_*) at the iteration k, and **M***_f_*(**p***_k_*) is the merit function given by

(20) $$\frac{1}{2}R^{T}\;\left(p_{k}\right)\;R\;\left(p_{k}\right)$$

The derivative of the merit function at the iteration *k* is

(21) $${M^{\prime }}_{f}\left(p_{k}\right)=J^{T}\;\left(p_{k}\right)R\left(p_{k}\right)$$

Δ*_LM_* is the Levenberg–Marquardt direction,

(22) $$\mu_{k}=\rho \left\|J^{T}\;\left(p_{k}\right)R\left(p_{k}\right)\right\|$$

where ρ ∈ (0,1), and finally

(23) $$p_{0}=\frac{1}{M}\sum_{j=1}^{M}q_{j}$$

provides the initial guess required for the TRR and LM iterative algorithms.

**Algorithm 1:** Levenberg–Marquardt methodology.

**Require:** an initial position **p**_0_
**Ensure:** a solution **p**_*k*+1_
1: **Initialize:** k = 0,τ = Threshold, ρ = 0.05
2: **do**
3: Solve: (**J***^T^* (**p***_k_*)**J**(**p***_k_*) + *μ_k_***I**)Δ*_LM_* = *−***J***^T^* (**p***_k_*)**R**(**p***_k_*)
4: Find the sufficient decrease (Armijo’s condition):
5: such that $\alpha_{k}={\left(\frac{1}{2}\right)}^{t}$*for t* = 0*,*1*,...*
6: satisfies **M***_f_*(**p***_k_* + α*_k_*Δ*_LM_*) ≤ **M***_f_* (**p***_k_*) + 10*^−^*^4^α*_k_***M***^′^_f_*(**p**)*^T^* Δ*_LM_*
7: Update position: **p***_k_*_+1_ = **p** + α*_k_*Δ*_LM_*
8: Update *μ_k_*: *μ_k_* = ρ‖**J***^T^*(**p***_k_*)**R**(**p***_k_*)‖
9: **while**(‖**p**_*k*+1_ − **p***_k_*‖ ≤ τ or *k* ≤ 100)

## 4. A Bilateration Localization Method

In this section we present the bilateration method for WSN localization which can be used as the initialization step for iterative localization schemes. This algorithm avoids iterative procedures, gradient calculations, and matrix operations that increase the internal processing in a constrained device. This research was done independently of the work presented in [36]. Even though both schemes share the same idea (*i.e.*, bilateration), the procedure and the scope of both works are different as shown in Subsection 4.1. We show that it is possible to obtain a position estimate by solving a set of bilateration problems between a sensor node and its neighboring anchors, and then fusing the solutions according to the geometrical relationships among the nodes. Our aim is to find a scheme that can be deployed on a computationally constrained node. We argue that bilateration is an attractive option as the localization problem is divided on smaller sub-problems which can be efficiently solved on a mote. Next we start our development by introducing the typical assumptions and definitions considered in a WSN localization problem.

**Figure 1.**
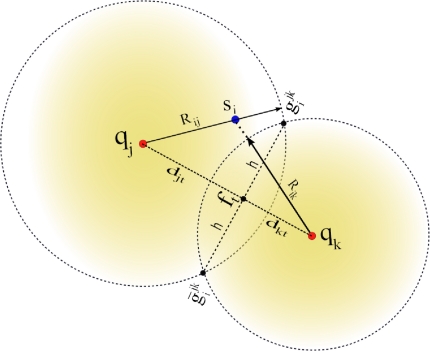
Sensor *s_i_* finding its two feasible solutions (**g***_i_*,**ḡ***_i_*) based on the anchors locations (**q***_k_*,**q***_j_*) and their respective anchor range measurements (*R_ij_*,*R_ik_*).

Let us define anchor subsets **A***_jk_* ⊂ **A** such that **A***_jk_* = {*a_j_, a_k_*} with *j* ≠ *k*. Hence, there is a total of $Q=\left(\underset{2}{M}\right)$ anchor subsets. Without loss of generality, consider the case for one node *s_i_* that receives from a subset *A_jk_* the anchor positions **q***_j_* and **q***_k_*, and computes the respective ranges *R_ij_* and *R_ik_* using RSS or ToA measurements. A possible geometrical scenario for this configuration is shown in Figure 1. We can appreciate from this example that the range estimates *R_ij_* is larger than *d_ij_* and *R_ik_* is shorter than *d_ik_*. Now, consider the two range circles shown in the figure; one with its origin at **q***_j_* and radius *R_ij_*, and the second with center in **q***_k_* and radius *R_ik_*. Next, define the two circle intersection points as $g_{i}^{\mathrm{jk}}$ and ${\bar{g}}_{i}^{\mathrm{jk}}$, where ${\bar{g}}_{i}^{\mathrm{jk}}$ is the reflection of $g_{i}^{\mathrm{jk}}$ with respect to the (imaginary) line that connects **q***_j_* and **q***_k_*. In this case, the superscript *jk* represents the anchor subset *A_jk_*. To simplify our discussion, we drop the superscripts, and only use them when more than one anchor subset is involved in our discussion.

In our approach, node *s_i_* determines the circle-circle intersections (*CCI*) **g***_i_* and **ḡ***_i_* by solving the closed-form expression reported in [37]. For instance, consider the two right triangles formed by the coordinates (**q***_j_*,**g***_i_*,**f***_t_*) and (**q***_k_*,**g***_i_*, **f***_t_*) in Figure 1, which satisfy the following relationships:

(24a) $$d_{\mathrm{jt}}^{2}+h^{2}=R_{\mathrm{ij}}^{2}$$

(24b) $$d_{\mathrm{kt}}^{2}+h^{2}=R_{\mathrm{ik}}^{2}$$

respectively. The distance *d_jt_* can be obtained by solving for *h*^2^ in Equations (24a) and (24b):

(25) $$R_{\mathrm{ij}}^{2}-d_{\mathrm{jt}}^{2}=R_{\mathrm{ik}}^{2}-d_{\mathrm{kt}}^{2}$$

and letting *d* = ‖**q***_j_* − **q***_k_*‖ = *d_jt_* + *d_kt_* resulting in

(26a) $$R_{\mathrm{ij}}^{2}-d_{\mathrm{jt}}^{2}=R_{\mathrm{ik}}^{2}-{\left(d-d_{\mathrm{jt}}\right)}^{2}$$

(26b) $$R_{\mathrm{ij}}^{2}=R_{\mathrm{ik}}^{2}-d^{2}+2\cdot d\cdot d_{\mathrm{jt}}$$

(26c) $$d_{\mathrm{jt}}=\frac{R_{\mathrm{ij}}^{2}-R_{\mathrm{ik}}^{2}+d^{2}}{2\cdot d}$$

where the position **f***_t_* = [*x_t_, y_t_*]*^T^* is obtained as follows:

(27) $$f_{t}=q_{j}+\frac{d_{\mathrm{jt}}}{d}\left(q_{k}-q_{j}\right)$$

Finally, the circle intersection **g***_i_* = [*x_i_,y_i_*]*^T^* is computed as

(28a) $$x_{i}=x_{t}\pm \frac{h}{d}\left(y_{k}-y_{j}\right)$$

(28b) $$y_{i}=y_{t}\mp \frac{h}{d}\left(x_{k}-x_{j}\right)$$

where **q***_j_* = [*x_j_,y_j_*]*^T^*, **q***_k_* = [*x_k_, y_k_*]*^T^*, and *h* is easily obtained from Equation (24). The complementary signs of Equations (28a) and (28b) are used to obtain the solution for **ḡ***_i_*.

**Figure 2.**
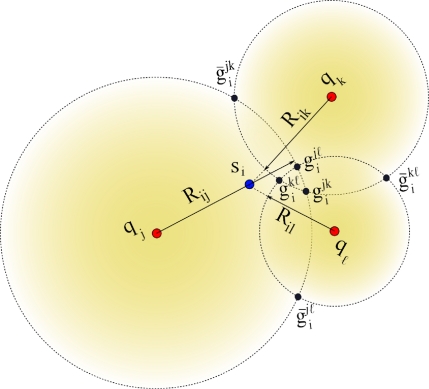
Sensor *s_i_* getting its initial estimation $P_{i}^{0}$ from three non-collinear anchors (*a_j_, a_k_, a_ℓ_*).

Each node *s_i_* applies the *CCI* procedure using all *Q* subsets **A***_jk_*. For instance, $g_{i}^{\mathrm{jk}}$ and ${\bar{g}}_{i}^{\mathrm{jk}}$ are obtained from the subset **A***_jk_*, $g_{i}^{j\ell }$ and ${\bar{g}}_{i}^{j\ell }$ are obtained from the subset **A***_jℓ_*, and so on. Hence, a sensor node will have 2*Q* possible initial position estimates where half are considered mirror solutions which should be eliminated through the selection process described next. Geometrically, we expect that the true location will be located around the region where solutions form a cluster (*i.e.*, half of the circle intersections should ideally intersect at the solution). Let us to consider the example shown in Figure 2.

There are three anchors named *a_j_, a_k_* and *a_ℓ_* and a node *s_i_* that needs to be localized. The range estimate *R_ij_* is larger than *d_ij_*, the range estimate *R_ik_* is shorter than *d_ik_*, and the range estimate *R_iℓ_* is shorter than *d_iℓ_*. Hence, *s_i_* computes a set of of six location candidates given by $\left\{g_{i}^{\mathrm{jk}},{\bar{g}}_{i}^{\mathrm{jk}},g_{i}^{j\ell },{\bar{g}}_{i}^{j\ell },g_{i}^{k\ell },{\bar{g}}_{i}^{k\ell }\right\}$. As seen in the figure, all the mirror circle intersection estimates will tend to be isolated while the correct circle intersections will tend to cluster around the node location. For example, to decide between $g_{i}^{\mathrm{jk}}$ and ${\bar{g}}_{i}^{\mathrm{jk}}$ candidate positions, generated using the anchors (*a_j_, a_k_*), the sensor *s_i_* obtains the minimum Square Euclidean sum from the location $g_{i}^{\mathrm{jk}}$ to each pair of candidate positions as follows:

(29) $$\psi =\text{min}\;\left({\left\|g_{i}^{\mathrm{jk}}-g_{i}^{j\ell }\right\|}^{2},{\left\|g_{i}^{\mathrm{jk}}-{\bar{g}}_{i}^{j\ell }\right\|}^{2}\right)+\text{min}\;\left({\left\|g_{i}^{\mathrm{jk}}-g_{i}^{k\ell }\right\|}^{2},{\left\|g_{i}^{\mathrm{jk}}-{\bar{g}}_{i}^{k\ell }\right\|}^{2}\right)$$

On the other hand, the sensor *s_i_* also obtains the minimum Square Euclidean sum from the location ${\bar{g}}_{i}^{\mathrm{jk}}$ to each pair of candidate positions as follows:

(30) $$\varphi =\text{min}\;\left({\left\|{\bar{g}}_{i}^{\mathrm{jk}}-g_{i}^{j\ell }\right\|}^{2},{\left\|{\bar{g}}_{i}^{\mathrm{jk}}-{\bar{g}}_{i}^{j\ell }\right\|}^{2}\right)+\text{min}\;\left({\left\|{\bar{g}}_{i}^{\mathrm{jk}}-g_{i}^{k\ell }\right\|}^{2},{\left\|{\bar{g}}_{i}^{\mathrm{jk}}-{\bar{g}}_{i}^{k\ell }\right\|}^{2}\right)$$

Finally, the lowest value of ψ and φ helps to decide between choosing $g_{i}^{\mathrm{jk}}$ or ${\bar{g}}_{i}^{\mathrm{jk}}$. The process is repeated for all *Q* solution pairs to generate a set of disambiguated locations.

**Algorithm 2:** General code used by every sensor *s_i_* to get its initial position estimate $p_{i}^{0}$.^1^

**Require: q***_k_*, *R_ik_*, *with* {*k* ← 1, …, *M*}, and $Q\leftarrow \left(\underset{2}{M}\right)$
**Ensure:**$p_{i}^{0}$
1: **Initialize: T** ← [0,0]*^T^*
2: **for** each subset $A_{\mathrm{jk}}\in \left(\underset{2}{M}\right)$ two-anchor subsets **do**
3: ψ ← 0
4: φ ← 0
5: $\left(g_{i}^{\mathrm{jk}},{\bar{g}}_{i}^{\mathrm{jk}}\right)$ ← *CCI* (**q***_j_*, **q***_k_*, *R_ij_*, *R_ik_*) {Return the two circle intersections}
6: **for** each subset $A_{\ell m}\neq A_{\mathrm{jk}}\in \left(\underset{2}{M}\right)$ two-anchor subsets **do**
7: $\left(g_{i}^{\ell m},{\bar{g}}_{i}^{\ell m}\right)$ ← *CCI* (**q***_ℓ_*, **q***_m_*, *R_iℓ_*, *R_im_*) {Return the two circle intersections}
8: $v_{1}\leftarrow {\left\|g_{i}^{\mathrm{jk}}-g_{i}^{\ell m}\right\|}^{2}$
9: $v_{2}\leftarrow {\left\|g_{i}^{\mathrm{jk}}-{\bar{g}}_{i}^{\ell m}\right\|}^{2}$
10: ψ ← ψ+min (*v*_1_,*v*_2_) {Return the minimum between *v*_1_ and *v*_2_}
11: $w_{1}\leftarrow {\left\|{\bar{g}}_{i}^{\mathrm{jk}}-g_{i}^{\ell m}\right\|}^{2}$
12: $w_{2}\leftarrow {\left\|{\bar{g}}_{i}^{\mathrm{jk}}-{\bar{g}}_{i}^{\ell m}\right\|}^{2}$
13: φ ← φ+min (*w*_1_,*w*_2_) {Return the minimum between *w*_1_ and *w*_2_}
14: **end for**
15: **if** (ψ < φ) **then**
16: $T\leftarrow T+g_{i}^{\mathrm{jk}}$
17: **else**
18: $T\leftarrow T+{\bar{g}}_{i}^{\mathrm{jk}}$
19: **end if**
20: **end for**
21: $p_{i}^{0}\leftarrow \frac{T}{Q}$

1 The algorithm omits the special cases where there are no circle intersections. The procedure for these instances is described further in the text.

Referring to our example, once node *s_i_* removes the mirror locations, then an estimate of the node position can be formed by taking the *average* of the disambiguated set $G=\left\{g_{i}^{\mathrm{jk}},g_{i}^{j\ell },g_{i}^{k\ell }\right\}$.

The complete bilateration scheme is described in Algorithm 2. This is a distributed localization algorithm in the sense that each node can implement it and determine its position estimate, given the anchor positions and the range estimates *R_ij_* between each node and all the anchors. Since Algorithm 2 uses only anchor measurement, it can be used as an initialization step to generate a set of position estimates that can be used with algorithms that integrate more information from anchor and non-anchor nodes (*i.e.*, iterative distributed algorithms).

**Figure 3.**
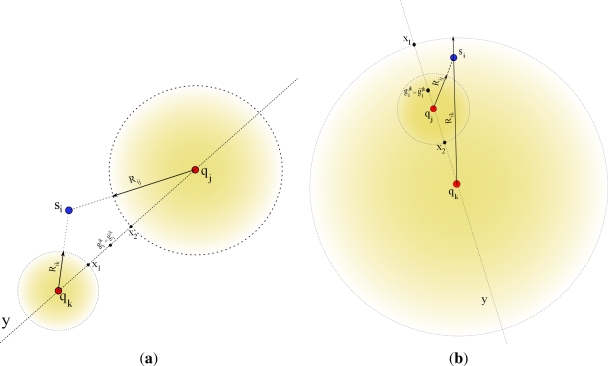
Possible cases where the triangle inequality is not satisfied. (**a**) Case A; (**b**) Case B.

There are some anomalous cases which should be considered in the bilateration algorithm. In order to get its initial estimation $p_{i}^{0}$, it is essential that every sensor *s_i_* gets the two location estimations from each one of the *Q* subsets even if the solutions are not feasible. For example, assume the two special cases shown in Figure 3. If we consider the left-side case on the figure, *R_ik_* is shorter than *d_ik_*, and *R_ij_* is shorter than *d_ij_*, clearly the triangle inequalities are not satisfied since

(31) $$\begin{array}{lll} R_{\mathrm{ij}}+R_{\mathrm{ik}} & > & \left\|q_{j}-q_{k}\right\|\\ R_{\mathrm{ij}}+\left\|q_{j}-q_{k}\right\| & > & R_{\mathrm{ik}}\\ R_{\mathrm{ik}}+\left\|q_{j}-q_{k}\right\| & > & R_{\mathrm{ij}} \end{array}$$

As a consequence, the sensor *s_i_* will not be able to find any solution to Equation (28). In other words, if the two circles do not intersect with each other, it will not be feasible to find the circle intersections **g***_i_* and **ḡ***_i_*. Therefore, a relaxed estimation should be generated as described next. Considering that ‖**q***_j_* − **q***_k_*‖ is a constant distance between the anchors in set **A***_jk_*, the node *s_i_* takes two steps to estimate the locations $g_{i}^{\mathrm{jk}}$ and ${\bar{g}}_{i}^{\mathrm{jk}}$. First, a location **x**_1_ is obtained by fixing *R_ik_* and making *R_ij_* = |‖**q***_j_* − **q***_k_*‖ − *R_ik_*| in order to satisfy the triangle inequality. Next, the sensor *s_i_* should use the *CCI* procedure to solve for **x**_1_. Similarly, a second location estimate **x**_2_ is obtained by fixing *R_ij_*, choosing *R_ik_* = |‖**q***_j_* − **q***_k_*‖ − *R_ij_*| to satisfy the triangle inequality and solving the problem through the *CCI* procedure. Finally, both $g_{i}^{\mathrm{jk}}$ and ${\bar{g}}_{i}^{\mathrm{jk}}$ are generated as the average $\frac{x_{1}+x_{2}}{2}$ implying that when the triangle inequality is not satisfied, there will be a single solution that falls over the line *y*. A similar procedure can be derived for the second case as depicted in Figure 3.

### 4.1. Comparison with the Previous Bilateration Scheme

As described before, the research reported in [36] is focused on a distributed bilateration scheme that finds initial estimates. Using two anchors at a time, each sensor node *s_i_* finds two possible candidates (*i.e.*, circle intersections). If sufficient anchors are available, the sensor node *s_i_* averages the cloud of candidates which tend to be close to each other. The average of such candidates provides the initial estimate.

As can be seen the general idea for this approach is quite similar to our bilateration approach. However, there are relevant differences between the two schemes that should be taken into account. These differences make that our bilateration approach be an alternative for the scheme proposed in [36]. For example, one of the differences is that [36] does not take into account special cases when a sensor *s_i_* is not able to compute circle intersections of two anchors (*i.e.*, the circles are not in touch) as shown Figure 3. Therefore, under this perspective this scheme is limited to naive scenarios in which estimated distances between sensors and anchors should have good accuracy. Thus, noisy RSS measurements, commonly used in realistic scenarios, may not provide useful information for this scheme. Hence, if a sensor *s_i_* is not able to find sufficient circle intersections from two neighboring pair of anchors at time, the localization process will fail. In our case, the bilateration scheme is able to obtain initial estimates under the most severe scenarios (*i.e.*, not circle intersections).

Another important aspect to consider in [36], is the use of a threshold, δ, which reduces the number of possible candidate positions, making this approach more selective. However, the value of δ is hard to determine in practice and also it does not guarantee good results in noisy environments. Additionally, in [36] each sensor node *s_i_* should create a table of its neighboring anchors. All anchors have a specific position inside of the table, and they are weighted by the sensor *s_i_* according to the candidate positions that they generate. The value of δ is used to select a certain group of candidate positions. The anchors are weighted according to the candidates that they generated. Finally, all tables are broadcast by sensors. Once all sensors have received the anchor tables of their neighbors, they run a post-processing stage to determine which anchors are more reliable than others. These anchors are used to obtain initial estimates. As can be seen, the drawback of this approach are extra wireless transmissions required to share anchor tables among sensors. In our case we present an extension of the earlier BL algorithm which avoids any kind of wireless transmission with the goal to save energy. Finally, we should remark that we are using a sorting algorithm (lines 10,13, and 15–18 of the Algorithm 2) to determine initial positions. Analysis results shown in next section demonstrate that the alternative BL algorithm is competitive in comparison with well known accurate and efficient algorithms based on least-squares methodologies.

## 5. Accuracy Performance Between Closed-Formulas and Iterative Procedures in the WSN Localization Problem

In this section we analyze the accuracy performance of both methodologies, optimized and closed-formula schemes. Even though the strength of a closed-formula for solving the WSN localization problem is its low complexity compared with an iterative algorithm, closed-formulas can present large errors in the presence of inaccurate ranging measurements. However, in many cases it is desirable to sacrifice accuracy to save energy (*i.e.*, increase battery lifetime). On the other hand, the weakness for closed-form methods (*i.e.*, noise sensitivity) represents the strong point for iterative methods and vice versa. The goal of both methodologies seems to be in opposite directions. However, the main effort in WSN localization research is focused on developing an strategy that can join the strength of both methodologies to create an efficient algorithm that can save energy providing the best accuracy in the estimated positions.

Next we present an evaluation of accuracy between closed-formulas and iterative methodologies. For the former methods we are considering the classical LS Multilateration, the Min-max method (The Min-max approach is based on the intersection of rectangles instead of circle intersections. It provides a more simple technique than lateration schemes to obtain position estimates at the expenses of accuracy) [38,39], and the bilateration algorithm. For iterative methodologies we are considering two algorithms to solve the NLLS: the LM and the TRR algorithms.

**Figure 4.**
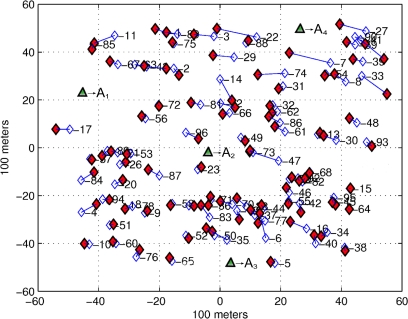
A typical WSN composed by 96 unknown sensors with true positions=‘⋄’, 4 non-collinear anchors =‘▴’, and 96 initial estimates=‘♦’. Each unknown sensor, using an initialization algorithm, estimates its initial position by using four reference positions (*A*_1_, *A*_2_, *A*_3_, and *A*_4_) and their respective estimated distances.

For the simulations that follow, we consider 20 different sensor networks where each one is composed by *N* = 96 unknown sensors, randomly distributed over 100 m by 100 m area. Also, we add *M* = 4 non-collinear anchors with full-connectivity on every realization as shown in Figure 4.

For each network, we add noise to the true distances between anchors and nodes using the log-distance path loss model described in Equation (4). The estimated distances are simulated using σ*_SH_* = 6*_dB_* and η*_P_* = 2.6, typical parameters for the propagation models on outdoors scenarios, and *P*_0_ = *−*52*_dB_* is selected according to current commercial specifications for wireless motes [40]. Finally, we assume that all nodes have the sensitivity to detect any RF signal coming from anchors.

**Figure 5.**
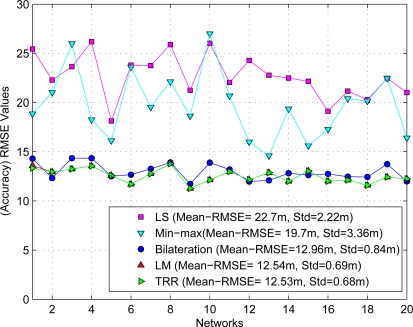
Position estimates provided by different initialization algorithms.

To compare the accuracy performance between both methodologies it was necessary to use the same set of range measurements for each closed-form method and iterative algorithm. Figure 5 summarizes the initial estimates obtained by both methodologies using the RMSE metric as shown the next equation:

(32) $$\mathrm{RMSE}=\sqrt{\frac{1}{N}\sum_{i=1}^{N}\left\|p_{i}^{0}-z_{i}\right\|}$$

where $p_{i}^{0}$ represents an initial position estimate for a sensor *s_i_* and **z***_i_* its true position.

As can be seen, the closed-form LS approach provides the least accurate initial estimates (mean = 22.7 *m* and standard deviation = 2.22 *m*) compared with iterative algorithms as expected due to the noisy ranging measurements. In a similar way, the Min-max scheme also provides large errors (mean = 19.7 *m* and standard deviation = 3.36 *m*). On the other hand, we can appreciate that both iterative algorithms, the LM and the TRR, provide practically the best and similar results for initial estimates (mean = 12.54 *m* and standard deviation = 0.69 *m*) as expected, and finally the bilateration algorithm presents very acceptable initial estimates compared with the last two algorithms (mean = 12.96 *m* and standard deviation = 0.84 *m*). However, we should consider that the computational complexity for the LM and the TRR algorithms is significantly larger than the bilateration algorithm. This discussion will be expanded in the next section.

Also, we tested the SX, SI and GSLS algorithms [25] using the same set of networks. The estimated positions presented large errors under this scheme as indicated by [26]. Then, these results were disregarded in our analysis.

## 6. Computational Complexity Analysis between the Bilateration and the LM Algorithm

The efficiency of an algorithm can be described in terms of the time or space complexity [41]. Time complexity refers to the relation between the number of required computations for a given input. The space used for a given input provides the space complexity of an algorithm. The computational complexity of an algorithm could be described as the number of operations that it takes to find a solution [42].

In this section we provide an operation count on the number of additions (ADDs), multipliers (MULs), divides (DIVs), and square roots (SQRTs) exactly in the way that DSP algorithms are described [43]. This will allow an “apple-to-apple” comparison. Moreover, an accurate description lends itself to a cycle accurate description for any microprocessor and more significantly, the use of energy models based on computing cycles to estimate the energy consumption for a given algorithm. An energy analysis will be a discussion of a future work. In next subsections we present the computational complexity analysis for the iterative LS and the bilateration algorithm.

### 6.1. Computational Analysis of the LM Algorithm

The LM algorithm could be considered as too expensive for motes given its iterative nature and the need to estimate first and second order information (*i.e.*, gradients, Jacobians, and Hessians). The number of iterations *K* is highly dependent on the initial point **x**_0_ and could be considered a random variable. On the other hand, if a good initial estimate, **x**_0_, is provided, then the number of iterations is expected to be low given the convergence properties of LM.

We are interested in providing an algorithmic analysis that provides a detailed description in terms of additions and subtractions (jointly referred as ADDs), multiplications (MULs), divisions (DIVs), and square roots (SQRTs). For simplicity in the next paragraphs we let **J***_k_* ≡ **J**(**x***_k_*) and **R***_k_* ≡ **R**(**x***_k_*).

The square root is a relevant operation as the error function **R***_k_* and the Jacobian estimate requires *ℓ*_2_ norms to compute distances between sensor and anchors. We also note that the complexity of the operations is not the same in terms of the processing resources (hardware and software) they take. Abusing notation we have

(33) ADD<MUL<DIV<SQRT

This analysis also focuses on the most efficient implementation in terms of the proper operation sequencing in order to favor reuse of terms (*i.e.*, avoid computing the same quantity twice).

We perform the analysis for a single iteration of the LM algorithm, and the total cost for each operation is multiplied by *K*. We also note that *K* can be modeled as a random variable; the usefulness of this approach is discussed later. We assume there are *M* anchors which have broadcast their position to all the nodes. Each node will run the LM algorithm to find its initial position as described in Subsection 3.2. We identify three core operations: *ℓ*_2_ or Euclidean norm, the error vector **R***_k_* and an estimate of **J***_k_*.

The *ℓ*_2_ norm will be used to compute the magnitude of the difference between two vectors **a**, **b** ∈ ℝ^2^ given by $\left\|a-b\right\|=\sqrt{{\left(a_{x}-b_{x}\right)}^{2}+{\left(a_{y}-b_{y}\right)}^{2}}$. This requires three ADDS, two MULs and one SQRT. The norm is used to compute **R***_k_* given in Equation (18) and to estimate the Jacobian as follows:

(34) $$J_{k}={\left[\begin{array}{cc} \frac{-\left(x_{1}-x_{k}\right)}{\sqrt{{\left(x_{1}-x_{k}\right)}^{2}+{\left(y_{1}-y_{k}\right)}^{2}}} & \frac{-\left(y_{1}-y_{k}\right)}{\sqrt{{\left(x_{1}-x_{k}\right)}^{2}+{\left(y_{1}-y_{k}\right)}^{2}}}\\ \vdots & \vdots \\ \frac{-\left(x_{M}-x_{k}\right)}{\sqrt{{\left(x_{M}-x_{k}\right)}^{2}+{\left(y_{M}-y_{k}\right)}^{2}}} & \frac{-\left(y_{M}-y_{k}\right)}{\sqrt{{\left(x_{M}-x_{k}\right)}^{2}+{\left(y_{M}-y_{k}\right)}^{2}}} \end{array}\right]}_{M\times 2}$$

For **R***_k_* we see that we require *M* ADDs and *M ℓ*_2_-norms. Accounting for the norms, the error function requires 4*M* ADDs, 2*M* MULs, and *M* SQRTs. These numbers are recorder in Table 1. A similar analysis follows for **J***_k_*. A direct look at Equation (34) indicates that we have the same norm across rows, so we can compute them first and then we would need an additional 2*M* ADDs and 2*M* DIVs. However, a better approach would be to compute the terms 1/‖**x***_j_ −* **x***_k_*‖ first so that we would require *M* DIVs, 2*M* MULs, and 2*M* ADDs. We exchange *M* DIVs by 2*M* MULs under the typical case that MULs have a much lower complexity than DIVs, particularly for the case of floating point operations. The complexity for the Jacobian estimate is also shown on Table 1.

Once these two quantities have been evaluated, their use trickles down through the algorithm. The costs for the different steps or operations is presented in the remaining part of Table 1. We just make two more remarks on the algorithm complexity. First, note that the approximation to the Hessian matrix $J_{K}^{T}\;J_{K}$

(35) $$\nabla^{2}f\left(x_{k}\right)=J_{k}^{T}\;J_{k}={\left[\begin{array}{cc} \sum_{j=1}^{M}\;\left(\frac{{\left(x_{j}-x_{k}\right)}^{2}}{\left({\left(x_{j}-x_{k}\right)}^{2}+{\left(y_{k}-y_{j}\right)}^{2}\right)}\right) & \sum_{j=1}^{M}\;\left(\frac{\left(x_{j}-x_{k}\right)\left(y_{j}-y_{k}\right)}{\left({\left(x_{j}-x_{k}\right)}^{2}+{\left(y_{j}-y_{k}\right)}^{2}\right)}\right)\\ \sum_{j=1}^{M}\;\left(\frac{\left(x_{j}-x_{k}\right)\left(y_{j}-y_{k}\right)}{\left({\left(x_{j}-x_{k}\right)}^{2}+{\left(y_{j}-y_{k}\right)}^{2}\right)}\right) & \sum_{j=1}^{M}\;\left(\frac{{\left(y_{j}-y_{k}\right)}^{2}}{\left({\left(x_{j}-x_{k}\right)}^{2}+{\left(y_{k}-y_{j}\right)}^{2}\right)}\right) \end{array}\right]}_{2\times 2}$$

is of size 2 × 2 which makes its inversion trivial when computing the LM step Δ*_LM_*, as shown in Algorithm 1.

(36) $$\Delta_{\mathrm{LM}}={\left(J_{k}J_{k}^{T}+\mu_{k}I\right)}^{-1}J_{k}^{T}R_{k}$$

where the gradient of the function $J_{k}^{T}R_{k}$ is given by

(37) $$\nabla_{f}\left(x_{k}\right)=J_{k}^{T}R_{k}={\left[\begin{array}{c} \sum_{j=1}^{M}\;\left(\frac{\left(x_{j}-x_{k}\right)\cdot \left(R_{\mathrm{kj}}-\sqrt{{\left(x_{j}-x_{k}\right)}^{2}+{\left(y_{j}-y_{k}\right)}^{2}}\right)}{\sqrt{{\left(x_{j}-x_{k}\right)}^{2}+{\left(y_{j}-y_{k}\right)}^{2}}}\right)\\ \sum_{j=1}^{M}\;\left(\frac{\left(y_{j}-y_{k}\right)\cdot \left(R_{\mathrm{kj}}-\sqrt{{\left(x_{j}-x_{k}\right)}^{2}+{\left(y_{j}-y_{k}\right)}^{2}}\right)}{\sqrt{{\left(x_{j}-x_{k}\right)}^{2}+{\left(y_{j}-y_{k}\right)}^{2}}}\right) \end{array}\right]}_{2\times 1}$$

Second, satisfying the sufficient decrease condition is also an iterative procedure where different values of α*_k_* are tested. We identify *T* as the number of iterations needed to satisfy this condition. As we discuss later, we will model *T* as a random variable.

The last row of the table provides the total which we identify as *T_ADD_*, *T_MUL_*, *T_DIV_* and *T_SQRT_* respectively. These numbers are the operations for a single iteration of the LM algorithm. Then, for *K* iterations we have the total number of operations to be *K_ADD_* = *K · T_ADD_*, *K_MUL_* = *K · T_MUL_*, *K_DIV_* = *K · T_DIV_*, and *K_SQRT_* = *K · T_SQRT_* .

Since the values of *T* and *K* are random variables, then a more convenient approach to quantify the number of operations would be to look at the average number of operations, *i.e.*, the expected value. It is intuitive to assume that *T* and *K* are independent, and that for a given network their distributions will be identical. Hence, we define

(38) $${\bar{K}}_{\mathrm{ADD}}=\varepsilon \left\{K_{\mathrm{ADD}}\right\}=\varepsilon \left\{K\right\}\varepsilon \left\{T_{\mathrm{ADD}}\right\}=\varepsilon \left\{K\right\}\left(\varepsilon \left\{T\right\}\left(M+4\right)+15M+7\right)$$

(39) $${\bar{K}}_{\mathrm{MUL}}=\varepsilon \left\{K_{\mathrm{MUL}}\right\}=\varepsilon \left\{K\right\}\varepsilon \left\{T_{\mathrm{MUL}}\right\}=\varepsilon \left\{K\right\}\left(\varepsilon \left\{T\right\}\left(M+2\right)+10M+15\right)$$

(40) $${\bar{K}}_{\mathrm{DIV}}=\varepsilon \left\{K_{\mathrm{DIV}}\right\}=\varepsilon \left\{K\right\}\left(M+1\right)$$

(41) $${\bar{K}}_{\mathrm{SQRT}}=\varepsilon \left\{K_{\mathrm{SQRT}}\right\}=\varepsilon \left\{K\right\}\left(2M+2\right)$$

where ε{*x*} represents the expected value of the random variable *x*. Finally, we can quantify the total complexity of the LM algorithm by converting operations to a common denominator and compute a single representative number that can be used for comparison with other algorithms. The typical way to quantify operations is to use the number of processor cycles (on the average) required to complete each type of operation. Let us define *N_ADD_*, *N_MUL_*, *N_DIV_*, and *N_SQRT_* as the number of cycles required for floating addition (or subtraction), a multiplication, a division, and square root, respectively. We should note that these numbers depend on the micro-processor and hardware used by the mote and the compiler tools used to develop the software. Hence, in practice the best way to obtain these values is through code profiling using a cycle-accurate simulator. Moreover, as discussed in [44], the number of task cycles can be used as part of models that measure energy consumption. Hence, as final measure of complexity for the LM algorithm we compute the total number of cycles as

(42) $$N_{\mathrm{LM}}=N_{\mathrm{ADD}}{\bar{K}}_{\mathrm{ADD}}+N_{\mathrm{MUL}}{\bar{K}}_{\mathrm{MUL}}+N_{\mathrm{DIV}}{\bar{K}}_{\mathrm{DIV}}+N_{\mathrm{SQRT}}{\bar{K}}_{\mathrm{SQRT}}$$

### 6.2. Computational Analysis of the Bilateration Algorithm

The bilateration algorithm is very simple and non-iterative. For *M* anchors, a sensor node picks $\left(\underset{2}{M}\right)$ pairs of sensors and computes the intersections of the imaginary circles around each anchor with a radius given between the anchor and the sensor node. These intersections are computed using geometry with a procedure described by Equations (24–28). Then, a cluster with half of the computed intersections is found, providing an indication of the area where the node position is located. The number of operations required to compute two intersections is presented in Table 2.

Since this process is repeated $Q=\left(\underset{2}{M}\right)$ times, then the final row reflects the total operations multiplied by this factor. As the intersections are computed, the search for the cluster is performed by Equations (29) and (30). Since there are 2*Q* intersections, we need to select the *Q* that cluster together (*i.e.*, eliminate mirrors). The clustering is based on looking at the distance between all possible pairs of intersections and selecting those that exhibit the closets distances among themselves. This requires the calculation of $S=\frac{2Q(2Q-1)}{2}$ squared norms, and the use of a clustering or sorting algorithm to find the smallest *Q* elements from the list of *S* norm values. Taking advantage of the structure of the location points (*i.e.*, the two intersections from the same anchor pair are not compared), we can expect an average complexity of *O*(*S*) sorting steps using an algorithm like Quickselect algorithm [45]. Hence the final computational cost for the bilateration algorithm is presented in Table 3.

As with the LM algorithm, we close this subsection by providing an expression in terms of processor cycles. Using the same characterization for all main operations of the algorithm, we can provide a total cycle count that can be directly compared with other algorithms. Obviously, a lower cycle implies lower complexity when the hardware and software development tools are identical. The expression for total cycles is

(43) $$N_{\mathrm{BL}}=N_{\mathrm{ADD}}\cdot \left(11Q+3S\right)+N_{\mathrm{MUL}}\cdot \left(12Q+2S\right)+N_{\mathrm{DIV}}.\left(3Q\right)+N_{\mathrm{SQRT}}\cdot \left(2Q\right)+N_{\mathrm{SORT}}$$

It is easy to see that the bilateration scheme uses a significantly less number of cycle for all operations. Experimental data in [45] indicates that the cycle count for the complete sorting step with the *QuickSelect* algorithm with a pipelined architecture can be achieved within 2500 and 3000 clock cycles.

To complete the computational complexity analysis, we need the number of CPU cycles required for the four basic operations as floating point operations. These values are highly dependent on the architecture of the mote processor. An extensive study in [46] provides good representative values for processors with some level of hardware support. The values are summarized on Table 4, and it shows the relation between basic operations and CPU cycles.

Next, we use Tables 1 and 2 to obtain the number of CPU cycles required by each initialization stage, BL and LM respectively. For the LM initialization stage we are using M = 4 anchors and the random variables *T* and *k*. We recall that *k* is the number of iterations spent by the LM algorithm to find a solution. These values are obtained through simulations where ε{*T*} = 2 and ε{*k*} = 13. In this way the total cycles required by the LM algorithm according to Equation (42) is given by

(44) $$\begin{array}{ll} K_{\mathrm{LM}} & =\varepsilon \left\{k\right\}\;[\left(\left(M+4\right)\varepsilon \left\{T\right\}+15M+7\right)\;\left(11\right)+\\ & \;\;\;\left(\left(M+2\right)\varepsilon \left\{T\right\}+12M+18\right)\;\left(25\right)+\\ & \;\;\;\left(M+1\right)\left(112\right)+\\ & \;\;\;\left(2M+4\right)\left(119\right)]=63063\;\text{cycles} \end{array}$$

Similarly, the total number of cycles used by the BL stage is given by Equation (43) as

(45) $$\begin{array}{ll} K_{\mathrm{BL}}= & \left(11\right)\left(11Q+3S\right)+\\ & \left(25\right)\left(12Q+2S\right)+\\ & \left(112\right)\left(3Q\right)+\\ & \left(119\right)\left(2Q\right)+\\ & \left(N_{\mathrm{SORT}}\right)=14198\;\text{cycles} \end{array}$$

where *Q* = 6*, S* = 66, and *N_SORT_* = 2750. The value for *N_SORT_* represents the total number of cycles required to perform the sorting step of the BL algorithm. This step can be performed using efficiently the Quickselect algorithm [47]. As expected, the LM algorithm consumes more energy in the initialization process than the BL scheme. However, the former represents a better choice when accuracy is required. Therefore, the BL can be an alternative localization scheme when a good tradeoff between accuracy and energy consumption is required on the initial estimates.

## 7. Conclusions

In this research, we analyzed a localization algorithm that can be realistically deployed over real WSNs which can provide good accuracy performance with low computational complexity. The bilateration algorithm is a distributed scheme that can be used as an initialization stage to find an initial set of locations.

Most initialization algorithms demand very high computing power to provide a set of initial estimates for an N-node WSN. The analyzed algorithm is capable to provide competitive initial estimates at low processing power. This approach is basically formed by two stages. The first stage consists of finding all circle intersections formed by anchor positions and their respective range estimates to a sensor node, obtained by ranging techniques like *ToA* or *RSS*. The great advantage of this approach is to use “closed-formulas” to find all circle intersections (*i.e.*, candidate positions) using two anchors at a time. In the second stage, the algorithm uses a sorting algorithm to find the cluster of candidate positions that tend to be closer to each other around the true location. The cluster with the nearby candidate positions is averaged to finally obtain the initial location. This scheme can be used by any WSN localization algorithm that needs initial approximations. Also, it is implementable in constrained devices with low processing and memory capabilities (*i.e.*, motes). Results show that this initialization algorithm is well behaved (e.g., computational and accuracy performance) in comparison with other well known algorithms like LS methodologies.

Finally, we are interested in exploring iteratively, at the refinement process, the Levenberg–Marquardt approach for node localization. We believe that this methodology can play a crucial role in producing excellent position estimates with high accuracy and low energy consumption due to the rate of convergence associated with this optimization technique.

## Figures and Tables

**Table 1.** LM Cost Functions.

**Title**	**ADD**	**MUL**	**DIV**	**SQRT**
**R***_k_*	4*M*	2*M*	0	*M*
**J***_k_*	5*M*	4*M*	*M*	*M*
**H***_k_*	3*M −* 3	3*M*	0	0
*M′_f_*	2*M−*2	2*M*	0	0
*M_f_*	*M−*1	*M* + 1	0	0
*μ_k_*	3	3	0	1
$H_{k}^{-1}$	3	6	1	0
Δ*_LM_*	2	4	0	0
Sufficient Decrease	*T* (*M* + 4)	*T* (*M* + 2)	0	0
Update	2	2	0	2
Stopping Condition	3	2	0	1

Total	(*M* + 4)*T* + 15*M* + 7	(*M* + 2)*T* + 12*M* + 18	*M* + 1	2*M* + 4

**Table 2.** Bilateration Cost Operations.

**Operations**	**ADD**	**MUL**	**DIV**	**SQRT**
*d*	3	2	0	1
*d_jt_*	1	5	1	0
*h*	1	1	0	1
$r=\frac{h}{d}$	0	0	1	0
**f***_t_*	2	2	1	0
**x***_i_*	1	1	0	0
**x̄***_i_*	1	0	0	0
**y***_i_*	1	1	0	0
**ȳ***_i_*	1	0	0	0
Total (2 circle intersections)	11	12	3	2
Total *Q* node combinations	11*Q*	12*Q*	3*Q*	2*Q*

**Table 3.** Final Computational Cost for the Bilateration Scheme.

**Action**	**ADD**	**MUL**	**DIV**	**SQRT**	**SORT**
Circle Intersections	11*Q*	12*Q*	3*Q*	2*Q*	0
Squared Norms	3S	2S	0	0	0
Number of Comparisons	0	0	0	0	*O*(*S*)

**Table 4.** Operation cycle counts.

**ADD**	**MUL**	**DIV**	**SQRT**
11	25	112	119
